# Using Project Extension for Community Healthcare Outcomes to Enhance Substance Use Disorder Care in Primary Care: Mixed Methods Study

**DOI:** 10.2196/48135

**Published:** 2024-04-01

**Authors:** MacKenzie Koester, Rosemary Motz, Ariel Porto, Nikita Reyes Nieves, Karen Ashley

**Affiliations:** 1 Weitzman Institute Moses Weitzman Health System Washington, DC United States

**Keywords:** continuing medical education, telementoring, substance use disorder treatment, substance use disorder, SUD, primary care, Extension for Community Healthcare Outcomes, Project ECHO

## Abstract

**Background:**

Substance use and overdose deaths make up a substantial portion of injury-related deaths in the United States, with the state of Ohio leading the nation in rates of diagnosed substance use disorder (SUD). Ohio’s growing epidemic has indicated a need to improve SUD care in a primary care setting through the engagement of multidisciplinary providers and the use of a comprehensive approach to care.

**Objective:**

The purpose of this study was to assess the ability of the Weitzman Extension for Community Healthcare Outcomes (ECHO): Comprehensive Substance Use Disorder Care program to both address and meet 7 series learning objectives and address substances by analyzing (1) the frequency of exposure to the learning objective topics and substance types during case discussions and (2) participants’ change in knowledge, self-efficacy, attitudes, and skills related to the treatment of SUDs pre- to postseries. The 7 series learning objective themes included harm reduction, team-based care, behavioral techniques, medication-assisted treatment, trauma-informed care, co-occurring conditions, and social determinants of health.

**Methods:**

We used a mixed methods approach using a conceptual content analysis based on series learning objectives and substances and a 2-tailed paired-samples *t* test of participants’ self-reported learner outcomes. The content analysis gauged the frequency and dose of learning objective themes and illicit and nonillicit substances mentioned in participant case presentations and discussions, and the paired-samples *t* test compared participants’ knowledge, self-efficacy, attitudes, and skills associated with learning objectives and medication management of substances from pre- to postseries.

**Results:**

The results of the content analysis indicated that 3 learning objective themes—team-based care, harm reduction, and social determinants of health—resulted in the highest frequencies and dose, appearing in 100% (n=22) of case presentations and discussions. Alcohol had the highest frequency and dose among the illicit and nonillicit substances, appearing in 81% (n=18) of case presentations and discussions. The results of the paired-samples *t* test indicated statistically significant increases in knowledge domain statements related to polysubstance use (*P*=.02), understanding the approach other disciplines use in SUD care (*P*=.02), and medication management strategies for nicotine (*P*=.03) and opioid use disorder (*P*=.003). Statistically significant increases were observed for 2 self-efficacy domain statements regarding medication management for nicotine (*P*=.002) and alcohol use disorder (*P*=.02). Further, 1 statistically significant increase in the skill domain was observed regarding using the stages of change theory in interventions (*P*=.03).

**Conclusions:**

These findings indicate that the ECHO program’s content aligned with its stated learning objectives; met its learning objectives for the 3 themes where significant improvements were measured; and met its intent to address multiple substances in case presentations and discussions. These results demonstrate that Project ECHO is a potential tool to educate multidisciplinary providers in a comprehensive approach to SUD care.

## Introduction

### Background

In the United States, overdose deaths continue to be a major cause of injury-related deaths. Since the onset of the COVID-19 pandemic, numbers have only accelerated, and the state of Ohio has led the nation in high substance use disorder (SUD) rates, including drug use and prescription drug use. The Centers for Disease Control and Prevention ranks the state among the top 5 across the United States with the highest rates of opioid overdose deaths [1]. While research has shown an increase in the number of people enrolled in substance use treatment in Ohio between 2015 and 2019 there was still a notable high increase in the annual average prevalence of past-year illicit drug use disorder in Ohio (3.6%) compared to the regional average (3%) and the national average (2.9%) [2]. In addition, past-month alcohol use disorder (9.3%), cannabis use disorder (5.8%), and tobacco use disorder (35.2%) were higher than the national average among young adults aged 18-25 years [2]. Ohio’s growing epidemic has highlighted the need to improve SUD care in a primary care setting by training providers to better address differences in care and social determinants of health through the use of behavioral techniques, harm-reduction philosophy of care, medication management, and a team-based care approach.

### Weitzman Extension for Community Healthcare Outcomes: Comprehensive Substance Use Disorder Care Program

Beginning in 2021, Buckeye Health Plan and Ohio University Heritage College of Osteopathic Medicine have partnered with the Weitzman Institute (WI), a national primary care research, policy, and education institute, to provide targeted support and education to Ohio primary care medical and behavioral health providers working with underserved patients, especially those in the rural, southeastern Appalachian region, using the evidence-based Project Extension for Community Healthcare Outcomes (ECHO) learning model. Project ECHO uses frequent videoconference sessions to connect a target audience of learners with subject matter experts for didactic and case-based instruction and engaged discussion [3]. Through regular attendance at these sessions, Project ECHO aims to equip learners with the knowledge, confidence, and skills to better manage complex cases.

WI has over 11 years of experience in developing and delivering Project ECHO programs to meet the needs of providers working in resource-limited settings. As an early adopter of the model in 2012, Weitzman ECHO programs have been offered in 22 topic areas to over 8000 health care professionals across all 50 states, Washington D.C., and Puerto Rico. Working in collaboration, Buckeye Health Plan and Ohio University aimed to leverage this expertise and offer multiple Project ECHO programs each year for providers in topics of the greatest need and interest.

As described, one of Ohio’s most dire population health needs is to improve outcomes for patients experiencing addiction. Thus, SUD was selected as the second ECHO program developed through this partnership. More specifically, opioids are a heightened concern throughout both Ohio and the United States, and the opioid epidemic has spurred significant funding allocations, such as the Biden Administration’s US $1.5 billion award to states and territories to end the epidemic [4]. However, there are many additional substances of concern, both illicit and nonillicit, such as alcohol, tobacco, cannabis, methamphetamine, and cocaine [5], which may receive less attention given the directed funding for opioids. For this reason, it was decided that the ECHO would address not only opioids, or any one substance, but rather be designed to provide techniques to help providers address SUD overall through a comprehensive, team-based lens and a harm reduction philosophy of care. Reflecting this broad topical approach, the program was titled the Weitzman ECHO: Comprehensive Substance Use Disorder Care (CSUDC ECHO) program.

CSUDC ECHO consisted of 24 twice-monthly sessions held between July 2021 and July 2022. Each 1-hour session included a 20- to 25-minute didactic presentation followed by 1 patient case submitted by a participant before the session and discussed live for the remaining 35-40 minutes. Textbox 1 outlines the didactic presentation topics for each session. A multidisciplinary core faculty facilitated each session and was comprised of 1 physician with dual board certification in family medicine and addiction medicine and experienced in treating SUDs at federally qualified health centers; 1 nurse practitioner who developed and leads a federally qualified health center medication-assisted treatment (MAT) program; 1 supervisory licensed counselor; and 1 population health expert. Together, the faculty built a 12-month curriculum covering diverse topics such as medication management, team-based care, trauma-informed care, stages of change and motivational interviewing, polysubstance use and co-occurring conditions, and coordinating levels of care.

**Weitzman Extension for Community Healthcare Outcomes: Comprehensive Substance Use Disorder Care didactic topic by session.**

**Session and didactic topic**

1. Philosophy of care (no case presentation).
2. Harm reduction strategies.
3. Principals of medication management.
4. Team-based care: care provision partners.
5. Trauma-informed care: an overview.
6. Motivational interviewing.
7. Stages of change for addiction.
8. Assessing stages of change and stage-based interventions.
9. Medications for opioid use disorder basics.
10. Behavioral health and primary care coordination.
11. Transitions of care.
12. Polysubstance use.
13. Social determinants of health including barriers or challenges (no case presentation).
14. Adverse childhood experiences.
15. Legal factors and access.
16. Mental health crisis and coordination of care.
17. Medication-assisted treatment for alcohol and tobacco use disorders.
18. Self-determination and strength-based approaches.
19. Contingency management for substance use disorder.
20. HIV and hepatitis C virus in patients with substance use disorder.
21. Screening, brief intervention and referral to treatment into primary care.
22. Stimulant use disorder treatment and medication management.
23. Co-occurring mental health substance use disorder.
24. Tobacco cessation for polysubstance patients.

Participants were recruited by email blasts targeted to each partner’s network of Ohio primary care providers and other members of the care team. A total of 109 participants attended at least one session, 16 participants attended between 7 and 11 sessions, and 23 participants attended over 12 (half) of the sessions. On average, there were 32 attendees at each session. Continuing education credits were offered to medical providers, behavioral health providers, and nurses.

### Purpose of Study

The purpose of this study was to assess the ability of CSUDC ECHO to both address and meet 7 learning objectives (Textbox 2) and address multiple substances by analyzing (1) the frequency of exposure to the learning objective topics and substance types during case discussions and (2) participants’ knowledge, self-efficacy, skills, and attitudes related to the treatment of SUDs pre- to postprogram.

**Weitzman Extension for Community Healthcare Outcomes: Comprehensive Substance Use Disorder Care learning objectives.**

- Project a harm reduction philosophy of care into your treatment of patients experiencing substance use disorders and explain this concept to peers.
- Use the care team more effectively to improve the management of patients experiencing substance use disorders.
- Use motivational interviewing and other behavioral techniques to improve patient outcomes related to substance use disorders.
- Better differentiate and implement medication management strategies for patients experiencing substance use disorders.
- Illustrate trauma-informed practices in the screening, assessment, and treatment of patients experiencing substance use disorders.
- Describe and manage common co-occurring conditions and polysubstance use more effectively in patients experiencing substance use disorders.
- Distinguish and address factors related to social determinants of health faced by specific populations experiencing substance use disorders.

## Methods

### Study Design and Data Collection

This study used a mixed methods design, using a conceptual content analysis [6] analyzing ECHO participant-led case presentations, as well as a 2-tailed paired-samples *t* test of participant self-reported learner outcomes. All ECHO attendees who registered and attended the Project ECHO CSUDC sessions are included in the deductive content analysis. All ECHO attendees who registered before and through the first session of the series were invited to complete a preseries survey (n=106) via Qualtrics survey software (Qualtrics). The preseries survey remained open for 3 weeks from June 25, 2021, to July 18, 2021. A total of 79 responses were received (n=79) for a response rate of 75%. Upon completion of the ECHO series, active attendees (ie, those that were still active at the conclusion of the series and did not officially drop from the series, as well as those who enrolled throughout the series) were invited to complete a postseries survey via Qualtrics Survey Software (n=90). The postseries survey remained open for 4 weeks from July 7, 2022, to August 2, 2022. A total of 25 responses were received (n=25) for a response rate of 28%. A total of 16 consented participants completed both the preseries and postseries surveys (n=16) and are included in the paired-samples *t* tests statistical analysis.

### Ethical Considerations

This study was approved by the Community Health Center, Inc, Institutional Review Board (IRB; 1190) on January 6, 2022. Informed consent was accounted for by the authors through the administration of a consent form on the postseries survey gathering participant consent to use their deidentified survey data for the paired-samples *t* test analysis. The deductive content analysis was considered a secondary analysis and was given exempt status. All data used in this study were deidentified, accounting for privacy and confidentially. No compensation for participation in this study was deemed necessary by the IRB.

### Survey Tools

The preseries and postseries surveys were internally created and based on the Consolidated Framework for Implementation Research (CFIR) [7] and Moore’s Model of Outcomes Assessment Framework [8]. The specific CFIR domains assessed for include intervention characteristics, outer setting, inner setting, characteristics of individuals, and process [7]. Additionally, the levels of Moore’s Model of Outcomes Assessment Framework assessed for include level 2 (satisfaction), level 3a (declarative knowledge), level 3b (procedural knowledge), level 4 (competence), level 5 (performance), and level 6 (patient health) [8]. The surveys assessed changes in participants’ self-reported knowledge, attitudes, self-efficacy, and skills through statements centered on the series’ learning objectives. The preseries survey also collected participant characteristics including provider type and years of experience working with patients diagnosed with SUDs, as well as team-based care practices. Additionally, the postseries survey collected information on engagement and practice changes. The preseries survey instrument is presented in Multimedia Appendix 1 and the postseries survey instrument is presented in Multimedia Appendix 2.

While the preseries survey and postseries survey tools were based on CFIR [7] and Moore’s Model of Outcomes Assessment Framework [8], both surveys were internally designed. The internal research and evaluation and CSUDC ECHO programmatic teams created the survey tools through several iterations of the internal review, which also consisted of selecting the appropriate domain (ie, knowledge, attitudes, self-efficacy, and skills) to assess each series’ learning objective. Each domain used a 5-point Likert scale to assess responses. The surveys were then presented to the CSUDC ECHO series stakeholders and faculty for review and approval before administering the surveys to the ECHO attendees. See Multimedia Appendices 1 and 2 for the domain placement of learning objectives and the 5-point Likert scales.

### Conceptual Content Analysis

To further evaluate Weitzman ECHO CSUDC aims, researchers conducted a conceptual content analysis [6] using a set of a priori themes extracted from the series’ learning objectives. Series’ learning objectives are detailed in Textbox 2. To establish a priori themes, researchers met before the launch of the ECHO to examine the series’ 7 learning objectives and extracted 7 themes for the content analysis. The themes were: harm reduction, team-based care, behavioral techniques, MAT, trauma-informed care, co-occurring conditions, and social determinants of health. To assess the frequency to which multiple substances were discussed, the themes also included 5 illicit and nonillicit substances of concern: alcohol, stimulants, opioids, cannabis, tobacco, or nicotine, plus polysubstance use when any 2 or more of these substances were identified. A conceptual analysis approach was used to gauge the dose and frequency of all learning objective themes and selected illicit and nonillicit substances. The content analysis aimed to confirm the discussion of the series’ learning objectives during case presentations and to determine to what extent multiple substances were able to be addressed.

Researchers evaluated all 22 participant-led ECHO case presentations and discussions for the presence of the selected themes in the prepared participant cases, faculty recommendations, and participant recommendations. Case presentations and discussions consisted of participants independently preparing a patient case to present and receive participant and faculty guidance for a patient treatment plan. Case presentations were recorded and transcribed using Zoom videoconferencing software (Zoom Video Communications, Inc). The transcriptions were then used for the conceptual content analysis.

To ensure coding accuracy, 4 researchers independently coded 27% (n=6) of the case presentations and met to reconcile discrepancies and better establish coding parameters. After reconciling discrepancies, 1 researcher coded the remaining 16 case presentations and discussion transcripts. The content analysis themes and descriptions are presented in Table 1.

**Table 1:** Conceptual content analysis themes and learning objectives.

Theme	Learning objective
Harm reduction	Project a person-centered philosophy of care into your treatment of patients experiencing substance use disorders and explain this concept to peers.
Team-based care	Use the care team more effectively to improve the management of patients experiencing substance use disorders.
Behavioral techniques	Use motivational interviewing and other behavioral techniques to improve patient outcomes related to substance use disorders.
Medication-assisted treatment	Differentiate and implement medication management strategies for patients experiencing substance use disorders.
Trauma-informed care	Illustrate trauma-informed practices in the screening, assessment, and treatment of patients experiencing substance use disorders.
Co-occurring conditions	Describe and manage common co-occurring conditions and polysubstance use more effectively in patients experiencing substance use disorders.
Social determinants of health	Distinguish and address factors related to social determinants of health faced by specific populations experiencing substance use disorders.

### Paired-Samples t Test

To determine if Project ECHO CSUDC affected participant learner outcomes, researchers calculated mean scores reported on a Likert scale of 1 to 5 and conducted a paired-samples *t* test to compare pre- and postseries scores at a .05 significance level. The surveys consisted of matching statements assessing knowledge, self-efficacy, attitudes, and skills associated with the series’ learning objectives. The data were assessed for normality and homogeneity of variance and the assumptions were met. The data analysis was conducted using SPSS Statistics for Windows (version 26.0; IBM Corp).

## Results

### Participant Characteristics

CSUDC ECHO participants were asked to report their role type on the preseries survey. Of the participants that responded to the survey items (n=79), a majority were other care team members (n*=*32; 41%) followed by behavioral health providers (n*=*30; 38%) and medical providers (n*=*16; 21%). Additionally, participants were asked to indicate their years of experience working with SUDs. Most participants had between 1 and 5 years of experience (n=23; 29%) followed by 6-10 years (n=15; 19%), 11-20 years (n=14; 18%), less than 1 year (n=13; 16%), 7 participants indicated they do not work directly with patients (n=7; 9%), 21-30 years (n=4; 5%), 31-40 years (n=2; 3%), and more than 40 years of experience (n=1; 1%). Full participant characteristics of the entire CSUDC ECHO attendees, excluding the paired-samples *t* test sample, the paired-samples *t* test sample only, and all combined CSUDC ECHO attendees are provided in Table 2.

The attendance data of participants included in the paired-samples *t* test analysis were analyzed. Further, 6 (n=6; 38%) of the paired-samples *t* test participants attended 1% (n=1) to 25% (n=6) of the 24 CSUDC ECHO sessions, 3 (n*=*3; 19%) attended 26% (n=7) to 49% (n=11) of the sessions, 4 (n*=*4; 25%) attended 50% (n=12) to 75% (n=18) of the sessions, and 3 (n*=*3; 19%) attended 76% (n=19) to 100% (n=24) of the sessions.

**Table 2:** Participant characteristics of all ECHO^a^ participants and paired-samples *t* test analysis sample.

			CSUDC^b^ ECHO attendees (excluding paired-samples *t* test participants; n=63)		Paired-samples *t* test participants (n=16)		All CSUDC ECHO attendees (n=79)
**Role type, n (%)**							
	Medical providers	13 (21)		3 (19)		16 (20)	
	Behavioral health providers	22 (35)		8 (50)		30 (38)	
	Other care team members	27 (43)		5 (31)		32 (41)	
	Missing	1 (2)		0 (0)		1 (1)	
**Years of SUD^c^ care experience, n (%)**							
	Less than 1	11 (17)		2 (13)		13 (16)	
	1-5	19 (30)		4 (25)		23 (29)	
	6-10	12 (19)		3 (19)		15 (19)	
	11-20	11 (17)		3 (19)		14 (18)	
	21-30	2 (3)		2 (13)		4 (5)	
	31-40	2 (3)		0 (0)		2 (3)	
	≥40	0 (0)		1 (6)		1 (1)	
	Does not work directly with patients	6 (10)		1 (6)		7 (9)	

^a^ECHO: Extension for Community Healthcare Outcomes.

^b^CSUDC: Comprehensive Substance Use Disorder Care.

^c^SUD: substance use disorder.

### Conceptual Content Analysis

The conceptual content analysis indicated that all of the a priori themes relating to the learning objectives resulted in high frequencies and doses, appearing in a majority of case presentations and discussions. Further, 3 themes appeared in 100% (n*=*22) of case presentations and discussions, including team-based care at a frequency of 156, followed by harm reduction at a frequency of 152, and social determinants of health at a frequency of 135. In total, 4 themes appeared in less than 100% (n=22) of case presentations and discussions, but above 81% (n=18), including co-occurring conditions with a frequency of 118 and appearing in 95% (n*=*21) of case presentations and discussions, followed by behavioral techniques at a frequency of 108 and appearing in 91% (n*=*20) of case presentations and discussions, MAT at a frequency of 89 and appearing in 86% (n*=*19) of case presentations and discussions, and trauma-informed care at a frequency of 79 and appearing in 82% (n=18) case presentations and discussions. Additionally, multiple substances were represented but at differing frequencies. The substance that resulted in the highest frequency and dose was alcohol at a frequency of 64 and appeared in 81% (n*=*18) of case presentations and discussions, followed by stimulants at a frequency of 55 and 77% (n=17) of case presentations and discussions, opioids at a frequency of 49 and 59% (n*=*13) of case presentations and discussions. Cannabis resulted with a frequency of 38 but appeared in 64% (n*=*14) of case presentations and discussions. Finally, tobacco and nicotine resulted in the lowest frequency at 11 and dose appearing in 27% (n*=*6) of case presentations and discussions. When evaluating polysubstance use, which was limited to the use of two or more of the listed substances, we found a dose of 95% (n*=*21) of case presentations and discussions. The frequency of polysubstance use was not included in the conceptual content analysis since it was not a learning objective theme and the emphasis of the conceptual content analysis was focused on the specific illicit and nonillicit substance types. The results of the conceptual content analysis are presented in Table 3.

**Table 3:** The results of frequency and percentage of case appearances (dose) of conceptual content analysis themes.

Theme	Frequency	Case appearances (dose), n (%)
Team-based care	156	22 (100)
Harm reduction	152	22 (100)
Social determinants of health	136	22 (100)
Co-occurring conditions	118	21 (95)
Behavioral techniques	108	20 (91)
MAT^a^	89	19 (86)
Trauma-informed care	79	18 (82)
Substance type: alcohol	64	18 (81)
Substance type: stimulant	55	17 (77)
Substance type: opioid	49	13 (59)
Substance type: cannabis	38	14 (64)
Substance type: tobacco and nicotine	11	6 (27)
Polysubstance use of substance types	—^b^	21 (95)

^a^MAT: medication-assisted treatment.

^b^—: not available.

### Paired-Samples t Test

#### Knowledge

In total, 4 knowledge domain statements resulted in statistically significant increases: understanding polysubstance use in patients experiencing SUD (*P*=.02), understanding the approach colleagues in other disciplines use to address SUD (*P*=.02), knowledge of medication management strategies for nicotine use disorder (*P*=.03), and knowledge of medication management strategies for opioid use disorder (OUD; *P*=.003). Additionally, all knowledge domain statements resulted in an increased change in mean score from preseries to postseries. The results of the knowledge domain preseries and postseries scores are presented in Table 4.

**Table 4:** The results of the paired-samples *t* test for the knowledge domain.

Statement	Preseries mean score (SD; 1-5)	Postseries mean score (SD; 1-5)	Change in mean	*P* value
I understand polysubstance use in patients experiencing substance use disorders	3.63 (1.03)	4.25 (0.45)	+0.62	.02
I understand factors related to social determinants of health faced by specific populations experiencing substance use disorders	4.13 (0.89)	4.31 (0.60)	+0.18	.38
I understand the approach of my colleagues in other disciplines (ie, behavioral health if you are a medical provider) to substance use disorder care	3.69 (0.87)	4.25 (0.58)	+0.56	.02
Knowledge of the different medication management strategies for patients experiencing—nicotine use disorder	3.40 (1.12)	4.00 (0.76)	+0.60	.03
Knowledge of the different medication management strategies for patients experiencing—alcohol use disorder	3.53 (0.99)	4.00 (0.54)	+0.47	.07
Knowledge of the different medication management strategies for patients experiencing—stimulant use disorder	3.07 (0.10)	3.71 (0.83)	+0.64	.10
Knowledge of the different medication management strategies for patients experiencing—opioid use disorder	3.56 (0.96)	4.19 (0.83)	+0.63	.003

#### Attitudes

No attitudes domain statements resulted as statistically significant. All attitudes domain statements resulted in an increased change in mean score from preseries to postseries except the statement about a treatment plan for a patient experiencing an illicit SUD only being successful if abstinence is maintained, which resulted in a negative change in mean score. The negative change in mean score from preseries to postseries was the appropriate direction of change for alignment with promoting a harm reduction philosophy. The results of the attitudes domain preseries and postseries scores are presented in Table 5.

**Table 5:** The results of the paired-samples *t* test for the attitudes domain.

Statement	Preseries mean score (SD; 1-5)	Postseries mean score (SD; 1-5)	Change in mean	*P* value
It is important to practice a harm reduction philosophy when treating patients experiencing substance use disorders	4.60 (0.63)	4.73 (0.46)	+0.13	.43
Practicing a harm reduction philosophy in the treatment of patients experiencing substance use disorders leads to better patient outcomes	4.47 (0.83)	4.60 (0.63)	+0.13	.50
It is important to identify factors related to social determinants of health that patients experiencing substance use disorders may be facing	4.71 (0.61)	4.86 (0.36)	+0.15	.44
Addressing factors related to social determinants of health in the treatment of patients experiencing substance use disorders leads to better patient outcomes	4.69 (0.60)	4.81 (0.40)	+0.12	.50
A treatment plan for a patient experiencing an illicit substance use disorder has only been successful if abstinence is maintained	2.25 (1.39)	2.00 (1.27)	–0.25	.43

#### Self-Efficacy

In total, 2 self-efficacy statements resulted in statistically significant increases: choosing a medication management strategy for nicotine use disorder (*P*=.002) and alcohol use disorder (*P*=.02). Additionally, all self-efficacy domain statements resulted in an increased change in mean score from preseries to postseries. The results of the self-efficacy domain preseries and postseries scores are presented in Table 6.

**Table 6:** The results of the paired-samples *t* test for the self-efficacy domain.

Statement	Preseries mean (SD; 1-5)	Postseries mean (SD; 1-5)	Change in mean	*P* value
Providing trauma-informed care	3.20 (1.01)	3.67 (0.90)	+0.47	.15
Using motivational interviewing techniques	3.31 (1.08)	3.81 (0.83)	+0.50	.12
Creating SMART^a^ goals with patients	3.43 (1.09)	3.79 (0.80)	+0.36	.29
Managing co-occurring conditions	3.40 (1.30)	3.93 (1.16)	+0.53	.16
Choosing an appropriate medication management strategy for—nicotine use disorder	2.75 (1.49)	3.88 (1.13)	+0.13	.002
Choosing an appropriate medication management strategy for—alcohol use disorder	2.63 (1.19)	3.75 (1.28)	+1.12	.02
Choosing an appropriate medication management strategy for—stimulant use disorder	1.86 (1.46)	2.00 (1.00)	+0.14	.79
Choosing an appropriate medication management strategy for—opioid use disorder	3.67 (1.41)	3.78 (1.48)	+0.11	.76

^a^SMART: specific, measurable, achievable, relevant, timely.

#### Skill

In total, 1 skill domain statement resulted in a statistically significant increase: using the stages of change theory to provide stage-based interventions to patients experiencing SUDs (*P*=.03). Additionally, all skill domain statements resulted in an increased change in mean score from preseries to postseries. The results of the skill domain preseries and postseries scores are presented in Table 7.

**Table 7:** The results of the paired-samples *t* test for the skill domain.

Statement	Preseries mean (SD; 1-5)	Postseries mean (SD; 1-5)	Change in mean	*P* value
Screening patients experiencing substance use disorders for trauma	3.53 (1.13)	4.07 (0.96)	+0.54	.06
Using the stages of change theory to provide stage-based interventions to patients experiencing substance use disorders	3.06 (1.29)	3.69 (0.87)	+0.63	.03
Collaborating with peer support specialists when working with patients experiencing substance use disorders	3.33 (1.29)	3.93 (1.03)	+0.60	.06
Referring patients to a higher level of care, such as IOP^a^, if needed	3.79 (0.98)	4.36 (0.75)	+0.57	.06
Preventing drug overdose of my patients experiencing a substance use disorder	2.83 (1.27)	3.25 (0.97)	+0.42	.10

^a^IOP: intensive outpatient.

## Discussion

### Principal Findings

Ohio’s annual average prevalence of tobacco use, heroin use, use of prescription pain relievers, OUDs, illicit drug use disorder, and SUD have been higher compared to both regional and national averages [2]. Considering the need to address this public health concern, CSUDC ECHO was implemented to train Ohio providers and care team members in substance use care. CSUDC ECHO enhanced the Project ECHO work in this field by focusing content and learning objectives on a comprehensive, team-based lens and a harm reduction philosophy of care to address multiple illicit and nonillicit substances including opioids, alcohol, nicotine, cannabis, and stimulants. To assess the ability of the CSUDC ECHO program to meet its 7 program learning objectives (Textbox 2) and address multiple substances, this study analyzed (1) the frequency of exposure to learning objective themes and substance types during case presentations and discussions and (2) participating providers’ change in knowledge, attitudes, self-efficacy, and skills related to the treatment of SUDs.

Study results demonstrate that all 7 learning objectives were frequently addressed in the content of case presentations and discussions throughout the program, with team-based care being the most frequently mentioned, 3 objectives appearing in 100% (n=22) of case discussions (eg, team-based care, harm reduction, and co-occurring conditions), and all 7 objectives appearing in >81% (n=18) of all cases discussed. This may have resulted in the learner outcome improvement pre- to postprogram for multiple learner domains (eg, knowledge, self-efficacy, and skill) for the following themes: team-based care, MAT, polysubstance use, and behavioral techniques. No pattern emerged among the participants included in the paired-samples *t* test analysis exposure to didactic topics and changes in learner outcomes.

Alcohol, stimulants, opioids, cannabis, and nicotine were addressed in the content of case presentations and discussions throughout CSUDC ECHO with alcohol being the most frequently mentioned and most common substance appearing in cases, 4 substances appearing in >59% (n=13) of case discussions (eg, alcohol, stimulants, opioid, and cannabis), and all coded substances appearing in at least a quarter of cases. The dialogue about these substances during case discussions likely resulted in improvements to the following learner outcomes related to medication management: alcohol use disorder, OUD, and nicotine use disorder. Medication management of cannabis use disorder was not assessed in the pre- to postsurveys. Additionally, the didactic presentation topics that centered on alcohol, opioid, and nicotine use disorder resulted in a higher attendance rate with about 40% (n=6) to 50% (n=8) of the participants included in the paired-samples *t* test analysis attending the sessions, as compared to only 20% (n=3) of the aforementioned participant sample having attended the session centered on stimulant use disorder.

These findings indicate that the ECHO program’s content aligned with its stated learning objectives; met its learning objectives for the 3 themes where significant improvements were measured; and met its intent to address multiple substances in case presentations and discussions. While case presentations and discussions comprise from half to the majority of content in the sessions (30-35 minutes of a 60-minute session), content during sessions also includes faculty didactic presentations (20-25 minutes), which also addresses these 7 learning objectives and various substances but was not a part of the content analysis. Therefore, learner outcome improvements may also be a result of content addressed in didactic presentations.

While the Project ECHO model has been shown to be effective in training the primary care workforce [9], specifically on OUD [10,11] and addiction medicine [12,13], there has been no documentation, to our knowledge, of the ability of a team-based, comprehensive SUD and polysubstance-focused Project ECHO designed to improve learner outcomes (eg, knowledge, self-efficacy, and skills). Although Komaromy and colleagues [14] investigated the frequency of cases presented based on substance type in a comprehensive SUD-focused ECHO, a content analysis of the case presentation and discussion transcripts was not analyzed to either assess the frequency of substances or learning objectives. Furthermore, to our knowledge, this process has not been combined in a mixed method approach to compare learner outcomes with a content analysis to gauge the ability of an SUD-focused Project ECHO program to meet its stated learning objectives. Our results reported here align with this literature and expand to demonstrate that Project ECHO is a potential tool to effectively educate multidisciplinary providers in a comprehensive approach to SUD care.

### Strengths

This study has several strengths which promote the ability of the Project ECHO model in enhancing health care providers’ knowledge, self-efficacy, and skill associated with comprehensive SUD care. The focus of this study is unique as there is minimal research exploring the benefits and training ability of Project ECHO with a comprehensive SUD care focus. This study’s noteworthy strength is the use of a mixed methods design that presents a comprehensive evaluation correlating the content addressed in the case presentations and discussions to statistically significant learner outcomes to demonstrate how this telementoring continuing education series improved provider’s knowledge, skills, and self-efficacy to benefit participating providers and their practices.

### Limitations

This study faced several limitations during data collection and analysis. The first limitation of this study was the limited sample size and low response rate. There was a decline between the number of participants who completed the preseries survey and postseries survey, resulting in a low comparative sample, which restricted the options for statistical analysis. Another limitation was generalizability; the results of this Project ECHO are limited to the target audience of medical providers, behavioral health providers, and care team members from the state of Ohio, which is not a representative sample of broader populations nationally. Additionally, participants self-selected to take part in the Project ECHO series, which presents the potential for self-selection bias. Another limitation this study faced was the lack of available or reliable data on Project ECHO and its ability to meet learning objectives and address multiple substances through providers’ knowledge, self-efficacy, skill, and attitudes. Furthermore, self-reported data to assess knowledge and skills, and self-reported data in general, could present participant biases and is difficult to corroborate with outcomes. The use of internally designed survey instruments instead of using validated instruments presents as a limitation. In light of these limitations, future studies in this subject matter should include a larger data set. Additionally, future studies using a nested analysis approach might provide more insight into how the learning objective themes coincide with the various illicit and nonillicit substance types and would be a useful analysis to contribute to the knowledge base. Another recommendation for future studies in this subject matter should include a deeper analysis of attendance dose and exposure to didactic topics to better understand the impact on changes in learner outcomes. Future research with greater validity will contribute to the significant gaps in literature regarding this subject.

### Conclusions

The purpose of this research study was to assess the ability of CSUDC ECHO to both address and meet 7 learning objectives (Textbox 2) and address multiple substances by analyzing (1) the frequency of exposure to the learning objective topics and substance types during case presentations and discussions and (2) participants’ knowledge, self-efficacy, skills, and attitudes related to the treatment of SUDs from pre- to postprogram. The results of this study indicate that CSUDC ECHO was able to both address and meet its learning objectives while addressing multiple substances, as demonstrated by improvements in learner knowledge, self-efficacy, and skills. All learning objective themes resulted in high frequencies and doses, appearing in a majority of case presentations throughout the series. These promising results suggest that Project ECHO is a potential tool to educate primary care providers, behavioral health providers, and care team members in a comprehensive approach to SUD assessment and treatment through complex case discussions combined with didactic learning for certain settings. As Project ECHO programs continue to be established globally and existing programs strengthen, further research examining the model’s ability to achieve positive learning outcomes and factors that may contribute to these outcomes (eg, frequency of topic dose) is needed to confirm the outcomes in larger population samples, additional topics of focus, and other geographical settings.

## Appendix

Multimedia Appendix 1 Weitzman Extension for Community Healthcare Outcomes: Comprehensive Substance Use Disorder Care preseries survey instrument.

Multimedia Appendix 2 Weitzman Extension for Community Healthcare Outcomes: Comprehensive Substance Use Disorder Care postseries survey instrument.

## Abbreviations

CFIR: Consolidated Framework for Implementation Research
CSUDC: Comprehensive Substance Use Disorder Care
ECHO: Extension for Community Healthcare Outcomes
IRB: institutional review board
MAT: medication-assisted treatment
OUD: opioid use disorder
SUD: substance use disorder
WI: Weitzman Institute

## Data Availability

The data sets generated and analyzed during this study are not publicly available due to a portion of the data being deemed as exempt by the institutional review board and the institutional review board approving a waiver of informed consent for the exempt data, as well as the sensitive nature of the data, but are available from the corresponding author on reasonable request.
