# Supplementary material for: Using Project Extension for Community Healthcare Outcomes to Enhance Substance Use Disorder Care in Primary Care: Mixed Methods Study
Source: JMIR Med Educ. 2024 Apr 1;10:e48135. doi: 10.2196/48135 (PMC11019412; doi:10.2196/48135)
Supplement: Multimedia Appendix 1 [file mededu_v10i1e48135_app1.docx]

**Multimedia Appendix 1.** Weitzman ECHO: Comprehensive Substance Use Disorder Care preseries survey instrument.

Q1 First Name:

________________________________________________________________

Q2 Last Name:

________________________________________________________________

Q3 What is your National Provider ID (NPI)?

- NPI:________________________________________________
- I forgot my NPI
- I do not have an NPI

Q4 How many unique health care organizations do you see patients at?

- 0
- 1
- 2
- 3
- More than 3 (please specify): ________________________________________________

Q5 Please indicate the number of years you have been in practice.

________________________________________________________________

Q6 Please indicate the number of years you have been working with patients with substance use disorders.

- Less than 1 year
- 1-5 years
- 6-10 years
- 11-20 years
- 21-30 years
- 31-40 years
- 40+ years
- I do not work directly with patients

Q7 Approximately what percentage of your current patients have the following substance use disorders?

|  | Not Applicable |
| --- | --- |

|  | 0 | 10 | 20 | 30 | 40 | 50 | 60 | 70 | 80 | 90 | 100 |
| --- | --- | --- | --- | --- | --- | --- | --- | --- | --- | --- | --- |

| % of patients with a nicotine use disorder | 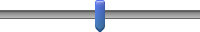 |
| --- | --- |
| % of patients with an alcohol use disorder | 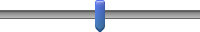 |
| % of patients with a stimulant use disorder | 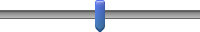 |
| % of patients with an opioid use disorder | 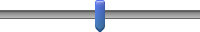 |

Q8 Please rate your knowledge of the different medication management strategies for patients experiencing:

|  | No knowledge (1) | Minimal knowledge (2) | Basic knowledge (3) | Adequate knowledge (4) | Superior knowledge (5) | N/A (6) |
| --- | --- | --- | --- | --- | --- | --- |
| Nicotine use disorder |  |  |  |  |  |  |
| Alcohol use disorder |  |  |  |  |  |  |
| Stimulant use disorder |  |  |  |  |  |  |
| Opioid use disorder |  |  |  |  |  |  |

Q9 Please rate your level of agreement with the following statements:

|  | Strongly disagree (1) | Disagree (2) | Unsure (3) | Agree (4) | Strongly agree (5) |
| --- | --- | --- | --- | --- | --- |
| I understand polysubstance use in patients experiencing substance use disorders. |  |  |  |  |  |
| I understand factors related to social determinants of health faced by specific populations experiencing substance use disorders. |  |  |  |  |  |
| I understand the approach of my colleagues in other disciplines (e.g., behavioral health if you are a medical provider) to substance use disorder care. |  |  |  |  |  |

Q10 Please rate your level of agreement with the following statements:

|  | Strongly disagree (1) | Disagree (2) | Unsure (3) | Agree (4) | Strongly agree (5) |
| --- | --- | --- | --- | --- | --- |
| It is important to practice a harm reduction philosophy when treating patients experiencing substance use disorders. |  |  |  |  |  |
| Practicing a harm reduction philosophy in the treatment of patients experiencing substance use disorders leads to better patient outcomes. |  |  |  |  |  |
| It is important to identify factors related to social determinants of health that patients experiencing substance use disorders may be facing. |  |  |  |  |  |
| Addressing factors related to social determinants of health in the treatment of patients experiencing substance use disorders leads to better patient outcomes. |  |  |  |  |  |
| A treatment plan for a patient experiencing an illicit substance use disorder has only been successful if abstinence is maintained. |  |  |  |  |  |

Q11 Please indicate how confident you are in choosing an appropriate medication management strategy for:

|  | Not at all confident (1) | Slightly confident (2) | Moderately confident (3) | Very confident (4) | Completely confident (5) | N/A (6) |
| --- | --- | --- | --- | --- | --- | --- |
| Nicotine use disorder |  |  |  |  |  |  |
| Alcohol use disorder |  |  |  |  |  |  |
| Stimulant use disorder |  |  |  |  |  |  |
| Opioid use disorder |  |  |  |  |  |  |

Q12 Please indicate how confident you are in the following:

|  | Not at all confident (1) | Slightly confident (2) | Moderately confident (3) | Very confident (4) | Completely confident (5) |
| --- | --- | --- | --- | --- | --- |
| Providing trauma-informed care |  |  |  |  |  |
| Using motivational interviewing techniques |  |  |  |  |  |
| Creating SMART goals with patients |  |  |  |  |  |
| Managing co-occurring conditions |  |  |  |  |  |

Q13 Please indicate your perception of your skill in the following:

|  | Not at all skilled (1) | Not very skilled (2) | Somewhat skilled (3) | Skilled (4) | Highly skilled (5) | N/A (6) |
| --- | --- | --- | --- | --- | --- | --- |
| Screening patients experiencing substance use disorders for trauma. |  |  |  |  |  |  |
| Using the Stages of Change Theory to provide stage-based interventions to patients experiencing substance use disorders. |  |  |  |  |  |  |
| Collaborating with peer support specialists when working with patients experiencing substance use disorders. |  |  |  |  |  |  |
| Referring patients to a higher level of care, such as Intensive Out-Patient (IOP), if needed. |  |  |  |  |  |  |
| Preventing drug overdose of my patients experiencing a substance use disorder. |  |  |  |  |  |  |

Q14 How often do you work with members of the care team in the following capacities:

|  | Never (1) | Rarely (2) | Sometimes (3) | Often (4) | Always (5) | N/A (6) |
| --- | --- | --- | --- | --- | --- | --- |
| I receive patient referrals from other care team members. |  |  |  |  |  |  |
| I communicate with other care team members through Electronic Health Records. |  |  |  |  |  |  |
| I discuss patients in rounds with one or more members present. |  |  |  |  |  |  |
| I work with other care team members to refer patients to a provider/care team member. |  |  |  |  |  |  |
| I work with other care team members to provide resources to patients on where they can receive additional care. |  |  |  |  |  |  |

Q15 What barriers do you face when working with patients experiencing substance use disorders?

________________________________________________________________

Q16 What is motivating you to participate in the ECHO? (check all that apply)

- I am interested in the topic
- I have patients who are experiencing a substance use disorder
- I want to improve my ability to treat patients experiencing a substance use disorder
- I want to receive Continuing Education Credits
- Others from my agency are participating
- My supervisor advised I participate in this
- Leadership at my agency advised that I participate in this ECHO

Other (please specify): ________________________________________________
