# Supplementary material for: Using Project Extension for Community Healthcare Outcomes to Enhance Substance Use Disorder Care in Primary Care: Mixed Methods Study
Source: JMIR Med Educ. 2024 Apr 1;10:e48135. doi: 10.2196/48135 (PMC11019412; doi:10.2196/48135)
Supplement: Multimedia Appendix 2 [file mededu_v10i1e48135_app2.docx]

**Multimedia Appendix 2.** Weitzman ECHO: Comprehensive Substance Use Disorder Care postseries survey instrument.

Q1 What two words or phrases would you use to describe Weitzman ECHO CSUDC?

________________________________________________________________

Q2 How would you rate the length of the series?

- Too short (I wish we had more sessions)
- Just right (The number of sessions held was perfect)
- Too long ( I would have preferred fewer sessions)

Q3 Please indicate your agreement with the following statements

|  | Strongly disagree (1) | Disagree (2) | Unsure (3) | Agree (4) | Strongly agree (5) |
| --- | --- | --- | --- | --- | --- |
| I actively participated in every ECHO session I attended. |  |  |  |  |  |
| I was free from distraction during every ECHO session I attended. |  |  |  |  |  |
| I have often referenced the materials and resources shared on this ECHO (e.g., PPT slides, recordings, etc.). |  |  |  |  |  |
| I often shared what I learned from this ECHO with my colleagues. |  |  |  |  |  |

Q4 How would you rate your overall satisfaction of the following:

|  | Very dissatisfied (1) | Dissatisfied (2) | Neutral (3) | Satisfied (4) | Very satisfied (5) |
| --- | --- | --- | --- | --- | --- |
| Support provided by Program Specialist |  |  |  |  |  |
| Knowledge gained from Faculty |  |  |  |  |  |
| Content covered during didactic presentations |  |  |  |  |  |
| Strategies discussed during case presentations |  |  |  |  |  |
| ECHO Learning Environment |  |  |  |  |  |

Q5 Please rate your knowledge of the different medication management strategies for patients experiencing:

|  | No knowledge (1) | Minimal knowledge (2) | Basic knowledge (3) | Adequate knowledge (4) | Superior knowledge (5) | N/A (6) |
| --- | --- | --- | --- | --- | --- | --- |
| Nicotine use disorder |  |  |  |  |  |  |
| Alcohol use disorder |  |  |  |  |  |  |
| Stimulant use disorder |  |  |  |  |  |  |
| Opioid use disorder |  |  |  |  |  |  |

Q8 Please rate your level of agreement with the following statements:

|  | Strongly disagree (1) | Disagree (2) | Unsure (3) | Agree (4) | Strongly agree (5) |
| --- | --- | --- | --- | --- | --- |
| I understand polysubstance use in patients experiencing substance use disorders. |  |  |  |  |  |
| I understand factors related to social determinants of health faced by specific populations experiencing substance use disorders. |  |  |  |  |  |
| I understand the approach of my colleagues in other disciplines (e.g., behavioral health if you are a medical provider) to substance use disorder care. |  |  |  |  |  |

Q6 Please rate your level of agreement with the following statements:

|  | Strongly disagree (1) | Disagree (2) | Unsure (3) | Agree (4) | Strongly agree (5) |
| --- | --- | --- | --- | --- | --- |
| It is important to practice a harm reduction philosophy when treating patients experiencing substance use disorders. |  |  |  |  |  |
| Practicing a harm reduction philosophy in the treatment of patients experiencing substance use disorders leads to better patient outcomes. |  |  |  |  |  |
| It is important to identify factors related to social determinants of health that patients experiencing substance use disorders may be facing. |  |  |  |  |  |
| Addressing factors related to social determinants of health in the treatment of patients experiencing substance use disorders leads to better patient outcomes. |  |  |  |  |  |
| A treatment plan for a patient experiencing an illicit substance use disorder has only been successful if abstinence is maintained. |  |  |  |  |  |

Q7 Please indicate how confident you are in choosing an appropriate medication management strategy for:

|  | Not at all confident (1) | Slightly confident (2) | Moderately confident (3) | Very confident (4) | Completely confident (5) | N/A (6) |
| --- | --- | --- | --- | --- | --- | --- |
| Nicotine use disorder |  |  |  |  |  |  |
| Alcohol use disorder |  |  |  |  |  |  |
| Stimulant use disorder |  |  |  |  |  |  |
| Opioid use disorder |  |  |  |  |  |  |

Q8 Please indicate how confident you are in the following:

|  | Not at all confident (1) | Slightly confident (2) | Moderately confident (3) | Very confident (4) | Completely confident (5) | N/A (6) |
| --- | --- | --- | --- | --- | --- | --- |
| Providing trauma-informed care |  |  |  |  |  |  |
| Using motivational interviewing techniques |  |  |  |  |  |  |
| Creating SMART goals with patients |  |  |  |  |  |  |
| Managing co-occurring conditions |  |  |  |  |  |  |

Q9 Please indicate your perception of your skill in the following:

|  | Not at all skilled (1) | Not very skilled (2) | Somewhat skilled (3) | Skilled (4) | Highly skilled (5) | N/A (6) |
| --- | --- | --- | --- | --- | --- | --- |
| Screening patients experiencing substance use disorders for trauma. |  |  |  |  |  |  |
| Using the Stages of Change Theory to provide stage-based interventions to patients experiencing substance use disorders. |  |  |  |  |  |  |
| Collaborating with peer support specialists when working with patients experiencing substance use disorders. |  |  |  |  |  |  |
| Referring patients to a higher level of care, such as Intensive Out-Patient (IOP), if needed. |  |  |  |  |  |  |
| Preventing drug overdose of my patients experiencing a substance use disorder. |  |  |  |  |  |  |

Q10 Please rate your level of agreement with the following statements:

|  | Strongly disagree (1) | Disagree (2) | Unsure (3) | Agree (4) | Strongly agree (5) |
| --- | --- | --- | --- | --- | --- |
| Participation in this ECHO has enhanced my professional satisfaction. |  |  |  |  |  |
| Participation in this ECHO has reduced my professional isolation. |  |  |  |  |  |

Q11 Where is your organization in the process of integrating behavioral health and primary care?

- Learning/exploring
- Beginning implementation
- Advanced/full implementation
- Ongoing quality improvement
- Other (please specify): __________________________________________________

Q12 How often do you work with members of the care team in the following capacities:

|  | Never (1) | Rarely (2) | Sometimes (3) | Often (4) | Always (5) | N/A (6) |
| --- | --- | --- | --- | --- | --- | --- |
| I receive patient referrals from other care team members. |  |  |  |  |  |  |
| I communicate with other care team members through Electronic Health Records. |  |  |  |  |  |  |
| I discuss patients in rounds with one or more members present. |  |  |  |  |  |  |
| I work with other care team members to refer patients to a provider/care team member. |  |  |  |  |  |  |
| I work with other care team members to provide resources to patients on where they can receive additional care. |  |  |  |  |  |  |

Q13 Please rate your level of agreement with the following statements:

|  | Strongly disagree (1) | Disagree (2) | Not Sure/Neutral (3) | Agree (4) | Strongly agree (5) |
| --- | --- | --- | --- | --- | --- |
| I apply best practices I have learned through this ECHO to all of my patients experiencing substance use disorders. |  |  |  |  |  |
| I have applied new team-based care strategies to my practice as a result of what I learned in this ECHO. |  |  |  |  |  |
| I am working to change clinical operations (e.g., policies, procedures, etc.) based on best practices learned in this ECHO. |  |  |  |  |  |
| I am working to provide a new clinical offering (e.g., a new clinical program, patient-focused activity, etc.) based on what I learned in this ECHO. |  |  |  |  |  |

Q14 Please list any changes you made because of what you learned in Weitzman ECHO CSUDC.
If you did not make any changes to these areas, please write "no changes".

- Changes in your individual practice:__________________________________________________
- Changes in your team-based care practice: _______________________________________________
- Changes in operations at your organization: ______________________________________________

Q15 Please list any barriers you faced while trying to make changes because of what you learned in Weitzman ECHO CSUDC. If you did not encounter barriers in these areas, please write "no barriers".

- Changes in your individual practice:__________________________________________________
- Changes in your team-based care practice: _______________________________________________
- Changes in operations at your organization: ______________________________________________

Q16 What ideas or solutions can you identify to help you overcome any of the barriers you listed above?
 Write "n/a" if you did not list barriers or "don't know" if you can't think of ideas or solutions.

________________________________________________________________

Q17 Please rate your level of agreement with the following statements:

|  | Strongly disagree (1) | Disagree (2) | Unsure (3) | Agree (4) | Strongly agree (5) |
| --- | --- | --- | --- | --- | --- |
| My patients' health outcomes have changed because I practiced what I learned in Weitzman ECHO CSUDC. |  |  |  |  |  |
| My patients’ health behaviors have changed because I practiced what I learned in Weitzman ECHO CSUDC. |  |  |  |  |  |

Q18 Please use this space to provide any additional feedback about your experience in this ECHO. (Optional)
